# Secreted β_3_-Integrin Enhances Natural Killer Cell Activity against Acute Myeloid Leukemia Cells

**DOI:** 10.1371/journal.pone.0098936

**Published:** 2014-06-11

**Authors:** Younis Skaik, Stefanie Vahlsing, Lilia Goudeva, Britta Eiz-Vesper, Anja Battermann, Rainer Blasczyk, Constança Figueiredo

**Affiliations:** Institute for Transfusion Medicine, Hannover Medical School, Hannover, Germany; UT MD Anderson Cancer Center, United States of America

## Abstract

Integrins are a large family of heterodimeric proteins that are involved in cell adhesion, migration, and proliferation. Integrin diversity and function is regulated by alternative splicing. Membrane-bound and truncated β_3_-integrins were shown to be key players in cancer metastasis. However, the immunomodulatory functions of the soluble (s) β_3_-integrin have not been investigated yet. In this study, we described a novel form of sβ_3_-integrin in acute myeloid leukaemia (AML) patients. Furthermore, we assessed the role of the sβ_3_-integrin in the modulation of natural killer (NK)-cell activity. Levels of sβ_3_-integrin were analysed in plasma samples of 23 AML patients and 26 healthy donors by ELISA. The capacity of sβ3-integrin to regulate NK cell activity was investigated using proliferation, cytokine secretion, and cytotoxicity assays. Circulating sβ_3_-integrin was detected in the plasma of 8 AML patients. NK cells showed significantly higher proliferation rates after stimulation with sβ_3_-integrin and IL-2, IL-15 (73%). Significant increases in the NK cells’ secreted levels of TNF-α, IFN-γ were measured in presence of sβ_3_-integrin. In addition, sβ_3_-integrin caused the upregulation of Granzyme B transcripts levels as well as FasL expression levels in NK cells. Most importantly, significantly higher K562 or AML blast target cell lysis rates were observed when NK cells were exposed to sβ_3_-integrin. This study reports the identification of a novel sβ_3_-integrin in AML patients and provides novel insights into its role in the immunomodulation of NK cell activity.

## Introduction

Integrins exist as obligate heterodimers receptors, which are constituted of an α and β transmembrane subunits. Each subunit consists of a large extracellular domain, a single-transmembrane domain, and a short cytoplasmic tail [1]. Integrins serve mainly as sensors for extracellular matrix ligands and cell surface ligands [2], [3]. Alternative splicing is an important mechanism to increase the functional diversity of integrins [4]. α_V_β_3_ and α_IIb_β_3_ integrins are constitutively expressed by angiogenic endothelial cells and platelets, respectively [5]. Although previous studies have shown that both membrane-bound and soluble (s) forms of β_3_-integrin are strongly associated with tumor cancer metastasis [6], [7], the immunomodulatory functions of the sβ_3-_ integrins remain unclear.

Acute myeloid leukemia (AML) is a frequent malignant hematological disease characterized by the initial accumulation of immature leukemia cells in the bone marrow and their subsequent migration into the blood circulation [8]. Natural Killer (NK) cells are key players in the immune surveillance of AML [9], and able to eradicate leukemic cells in an autologous or allogeneic setting [10], [11]. NK cell activity has been positively correlated with relapse-free survival after haematopoietic stem cell transplantation [12], [13]. NK cells use different strategies to eliminate their leukemic targets. NK cell-mediated clearance of leukemic cells may be induced by the secretion of perforins, granzymes and cytokines such as IFN- γ or TNF-α. Furthermore, NK cells are capable to upregulate the expression of Fas ligand (FasL, CD95L) to engage cell death receptors such as FAS/CD95 present on their target cells and thereby causing their apoptosis [14], [15], [16]. Previously, secretion of sβ_3_-integrin was demonstrated on human erythroleukemia (HEL) cells [17], however its role in the modulation of NK cell activity against leukemic blasts remained unclear. In this study we describe, for the first time to our knowledge, a novel sβ_3_-integrin variant in the plasma of AML patients. In addition, we have investigated the role of this alternative spliced sβ_3_-integrin on the immunomodulation of NK cell activity. Our results show that sβ_3_-integrin specifically enhances the cytotoxic activity of NK cells against leukemic target cells.

## Materials and Methods

### Patient and Control Samples

Plasma of 23 patients suffering of AML or secondary AML (sAML) following myelodysplastic syndromes (MDS) or without MDS antecedent (Table S1) was collected before and after chemotherapy. Informed written consent was obtained from all patients and approved by the local ethics committee of the Hannover Medical School. In addition, this study was also approved by the same committee and followed the principles expressed in the declaration of Helsinki. Twenty-six plasmas from healthy donors were used as controls. Plasmas from other myeloproliferative diseases (non-AML); acute lymphoblastic leukemia (ALL) (n = 1), chronic lymphoid leukemia (CLL) (n = 1), and (MDS) (n = 1), or non-Hodgkin lymphoma (NHL) (n = 1) were also collected prior and after chemotherapy.

### Detection of β_3-_integrin by ELISA

Maxisorp ELISA plates (Nunc, Wiesbaden, Germany) were coated with one-hundred µl undiluted or diluted plasma and incubated overnight at 4°C. Then, plates were washed with phosphate-buffered saline (PBS) and blocked with 2% BSA/PBST for 1 hour at room temperature (RT). After washing with PBS, 100 µL of the primary monoclonal antibody (MoAb) SZ-21 (Beckman Coulter, Marseille, France), CRC54 (antibodies-online GmbH, Aachen, Germany), Y2/51 (antibodies-online GmbH, Aachen, Germany), SZ-22 (Beckman Coulter, Marseille, France), P2 (Beckman Coulter, Marseille, France), or AP2 (GTI Diagnostic, Wisconsin, USA) were added to the plates previously pre-coated with plasmas. The specificity of each MoAb has been previously described [18]. After washing, 100 µL of the HRP-conjugate anti-mouse-IgG (Rockland Inc., Gilbertsville, PA, USA) were added to each well and incubated for 2 hours at RT. After addition of the 3,3′, 5,5′-Tetramethylbenzidin substrate (Dako, Hamburg, Germany), optical densities (OD) were measured at 450 nm using the TECAN Synergy (Tecan Deutschland GmbH, Crailsheim, Germany). Signal to noise values were calculated by subtracting the OD value of the blank to the OD value obtained in each test well. All samples were evaluated by blind testing. The cut-off value was calculated as the mean of OD values plus three standard deviations from the OD values obtained with the 26 negative control samples.

### RT-PCR, Vector Construction and Sequencing

Total RNA was isolated from the peripheral blood mononuclear cells (PBMCs) of the AML patients, using RNAase MiniKit (QIAGEN, Hilden, Germany) according to the manufacturer’s instructions. mRNA of each patient’s sample was reverse transcribed to cDNA using a multiscribe reverse transcriptase and Oligo dT (Applied Biosystems). All cDNA samples were subsequently stored at −20°C. Previously reported primers [6] were used to amplify β_3-_integrin from each patient and control cDNA sample. sβ_3-_integrin was amplified in PCR reactions using specific primers and the Bio-X-ACT short mix (Bioline, Luckenwalde, Germany). The thermal cycling conditions used were as follow: 94°C for 5 min, 30 cycles of 94°C for 20 sec, 60°C for 1 min, 72°C 2 min followed by a final extension of 72°C for 5 min. Fragments amplified during PCR were cloned into the plasmid pcDNA3.1 following the manufacturer’s instructions (Life technologies, Karlsruhe, Germany).

### Expression of Recombinant Soluble β_3-_integrin in HEK293

Soluble β_3-_integrin was produced recombinantly in HEK293 as previously described [19]. The recombinant sβ_3-_integrin sequence was designed based on the RNA sequence detected in the AML patients showing increased serum levels of sβ_3-_integrin. Therefore, the recombinant soluble β_3-_integrin is expected to have the same effect as the protein found in the AML patient’s serum. Briefly, a lentiviral vector encoding the sequence for sβ_3-_integrin was used for the HEK293 cells transduction in the presence of 8 µg/mL protamine sulphate (Sigma Aldrich Chemie, Munich, Germany). Seventy-two hours after transduction, cell culture supernatant was analyzed by ELISA for the presence of sβ_3-_integrin as previously described [19]. sβ_3-_integrin producing HEK 293 cells were cultured in bioreactors (*CELLine Adherent,* Integra, Fernwald, Germany) to increase the protein production yields. Cell-culture supernatants were harvested and stored at −20°C until the purification step.

### Purification of sβ_3-_integrin Using Immobilized Metal Affinity Chromatography

Soluble β_3-_integrin V5/His-tagged was purified from the cell-culture supernatant (pH 8.0) using HisTrap HP columns (Sigma-Aldrich Chemie, Steinheim, Germany), and the BioLogic DuoFlow System (Bio-Rad, Hercules, USA). Concentrations of soluble β_3-_integrin were determined using the bicinchoninic acid protein assay kit (Perbio Science, Bonn, Germany) and confirmed by ELISA using anti-V5 and HRP-anti-His as the capture and detection antibodies, respectively. Known amounts of V5-His-tagged β_3-_integrin protein were used as the reference standard. Produced sβ_3-_integrin was controlled for quality controlled by sodium dodecylsulfate (SDS) and immunoblot as previously described [19].

### Natural Killer Cell Isolation

Isolation of NK cells from PBMCs of healthy donors was performed by magnetic cell separation using the human NK-cell isolation kit (Miltenyi Biotec, Bergisch Gladbach, Germany), according to the manufacturer’s instructions. NK cells were identified using phycoerythrin (PE)-conjugated anti-NKp46 (Beckman Coulter Marseille, France) antibodies, and analysed using a FACSCanto flow cytometer (BD Biosciences, Immunocytometry Systems, San Jose, Calif, USA). Purities of isolated NK cells were higher than 95% in all experiments.

### Culture and Stimulation of NK Cells

Freshly isolated NK cells were cultured in RPMI medium (BioWhittaker/Cambrex, Hess. Oldendorf, Germany) supplemented with 10% human AB serum (C.C. Pro, Neustadt, Germany). For stimulation, NK cells were cultured in presence of the stimulatory cytokine IL-2 (100 U/ml) alone or in combination with IL-15 (50 ng/ml), only recombinant sβ_3-_integrin (5 µg/ml), or a cocktail including the cytokines and sβ_3-_integrin at the indicated concentrations. Both recombinant cytokines were purchased from PeproTech Inc. (Rocky Hill, USA). Non-stimulated NK cells were used as negative control (NS). A control protein was used to assess the specificity of the sβ_3-_integrin effect on NK cells. This control protein is a truncated form of a killer-immunoglobulin-like receptor (trKIR) protein produced and isolated similarly to sβ_3-_integrin and denatured prior its use as negative control (NC) by incubation at 56°C for 1 hour.

### Immunofluorescence

NK cells (1×10^5^ cells) were cultured on gelatinised 8-well Lab-Tek slides (Nunc, Rochester, USA), fixed with Cytofix (BD Bioscience). Five µg of His-tagged sβ_3-_integrin protein were added to the culture for 8 h. After washing, protein binding was detected using an anti-His antibody. NK cells cultured in the absence of sβ_3-_integrin were used as control. The samples were mounted in ProLong Antifade kit (Molecular Probes) and analysed using the 40x magnification lens of an Olympus IX81 microscope system and the Cell∧M software (Olympus, Hamburg, Germany).

### Proliferation Assay

Freshly isolated NK cells were labelled with carboxyfluorescein diacetate succinimidyl ester (CFSE) purchased from Invitrogen (Vybrant CFSE Cell Tracer Kit, Carlsbad, USA) at a final concentration of 4 µM. CFSE-labelled NK cells were incubated under the described conditions. After 7 days, NK-cell proliferation was assessed based on CFSE dilution using flow cytometry.

### Phenotype and Cytokine Secretion Assay

For phenotype and cytokine secretion analysis, freshly isolated NK cells were incubated under the described conditions for 48 hours. Expression levels of CD95L were determined by flowcytometric analyses upon cell staining with phycoerythrin-conjugated antibodies purchased at Beckman Coulter. Supernatants of NK-cell cultures were then collected and analyzed for cytokine secretion using Luminex technology (MILLIPLEX Human Cytokine/Chemokine 14-plex panel, Millipore, Billerica, MA, USA; Luminex 200TM instrument, Invitrogen).

### Real-Time PCR

Total RNA was isolated using the RNeasy Mini Kit (Qiagen, Hilden, Germany) and reverse transcribed into cDNA using the high-capacity cDNA reverse transcriptase kit (Applied Biosystems, Carlsbad, USA). Five ng of RNA was used for each experiment. Quantitative Real Time PCR was performed using the Real time-PCR Master Mix and the OneStep Plus RT system (Applied Biosystem). Primers were designed to amplify transcripts of Granzyme B as previously described [20]. Expression data was normalized using the reference gene β-actin.

### NK Cell Cytotoxic Assays

Freshly isolated NK cells were cultured in the conditions described above. After 48 h, NK cells were exposed to CFSE- labelled K562 cells at an effector: target (E∶T) ratio of 5∶1 for 6 h. Target cell lysis was determined upon staining with 7-Actinomycin D (7-AAD) (Molecular probes) by flow cytometric analysis. In addition, NK cell cytotoxic assays against primary AML blasts were performed to determine the effect and specificity of sβ_3_-integrin in the lysis of primary leukemia cells. For this purpose, AML blasts were enriched to reach a cell purity by up to 85% by cell sorting (FACSAria, BD Biosciences, San Jose, CA) and used as target cells. NK cells were treated with Fc receptor blocking reagent (Miltenyi Biotec) and exposed to sβ_3_-integrin in presence or absence of IL-2 and an anti-β_3_-integrin antibody (10 µg/ml; BD Biosciences). NK cell cytotoxic assays were performed and analysed as described above.

### Statistical Analysis

Statistical differences among means of two groups were calculated using the t-test. Differences between more than 2 groups were calculated using ANOVA. Statistical analyses were calculated using the GraphPad Prism 5 software (GraphPad Software, San Diego, CA). P values of less than 0.05 were considered significant.

## Results

### Detection of Soluble β_3–_integrin in Sera of AML Patients

**Figure 1:**
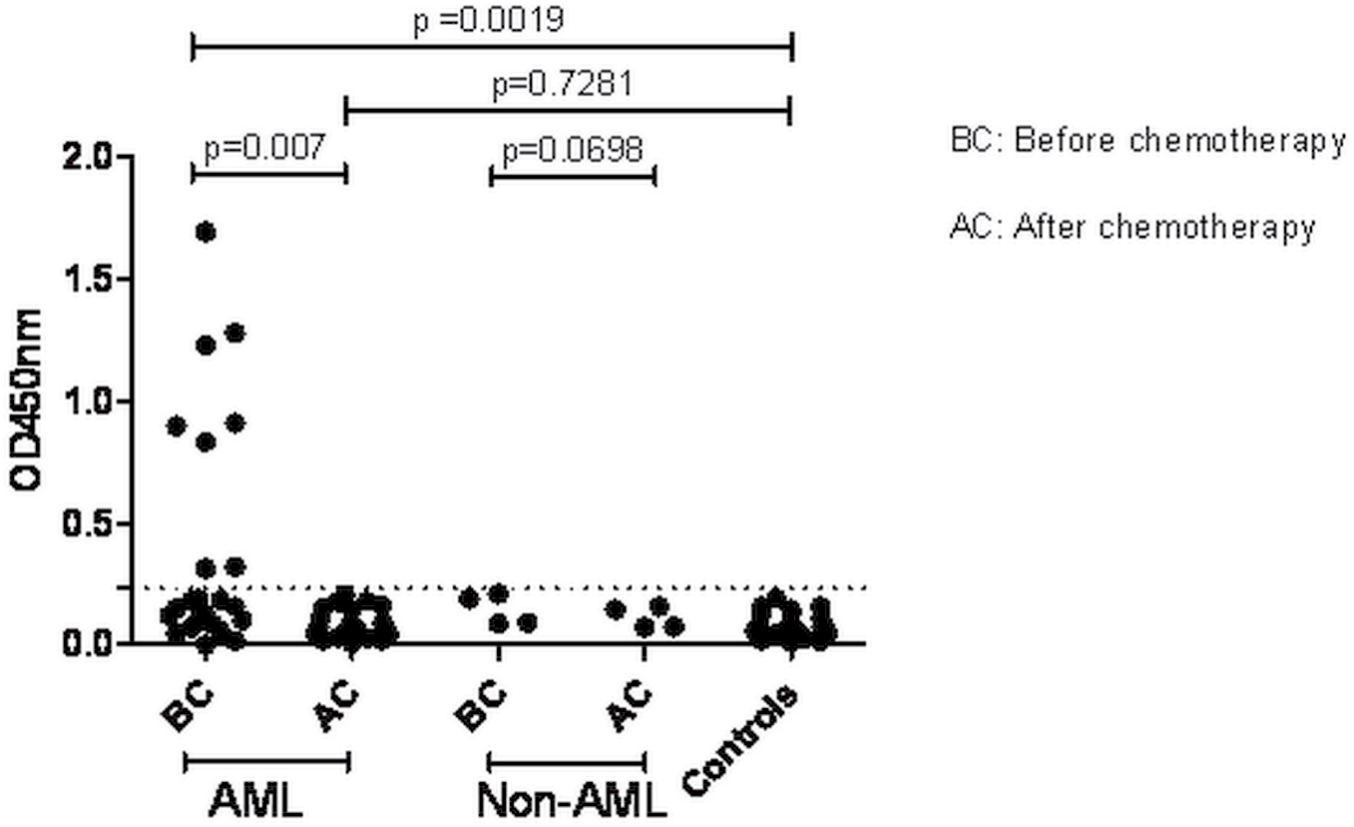
Detection of soluble β_3-_integrin in plasma of AML patients. Graph display sβ_3-_integrin optical density (ODs) in plasma obtained from normal donors (n = 26), non- acute myeloid leukemia (AML) patients (n = 4), and AML patients (n = 23) as obtained by ELISA. The dotted line represents the cut-off value 0.23 units of OD. Levels of significance were expressed as p-values (*p<0.05, **p<0.01, ***p<0.001).

Previously, we have established an ELISA for the detection of sβ_3–_integrin [19]. In this study, we have detected sβ_3–_integrin in 35% of AML patients (n = 8) prior chemotherapy. Significantly increased levels of sβ_3–_integrin were observed in AML patients in comparison to those detected after conditioning or in healthy individuals (p = 0.007, p = 0.0019, respectively). To confirm our findings, we used three different β_3_ integrin-specific murine MoAbs (SZ-21, CRC54, and Y2/51) and three control MoAbs (SZ-22, P2 and AP2) which recognize GPIIb alone, and the heterodimer GPIIb-IIIa, respectively. As expected, control MoAbs did not react with the sβ_3-_integrin (OD_450 nm_ range: 0.051–0.064) (Figure 1). Levels of sβ_3–_integrin were increased in patients showing different AML subtypes (M2 n = 2, M4 n = 1, M5 n = 2 and sAML n = 3) prior chemotherapy. No significant differences in sβ_3-_integrin levels were observed in non-AML patients prior or after chemotherapy (p = 0.0698) (Figure 1). These results suggest that the increase in sβ_3-_integrin levels is specific for AML patients.

### Identification of Alternatively Spliced Soluble Truncated β_3_ integrin

**Figure 2:**
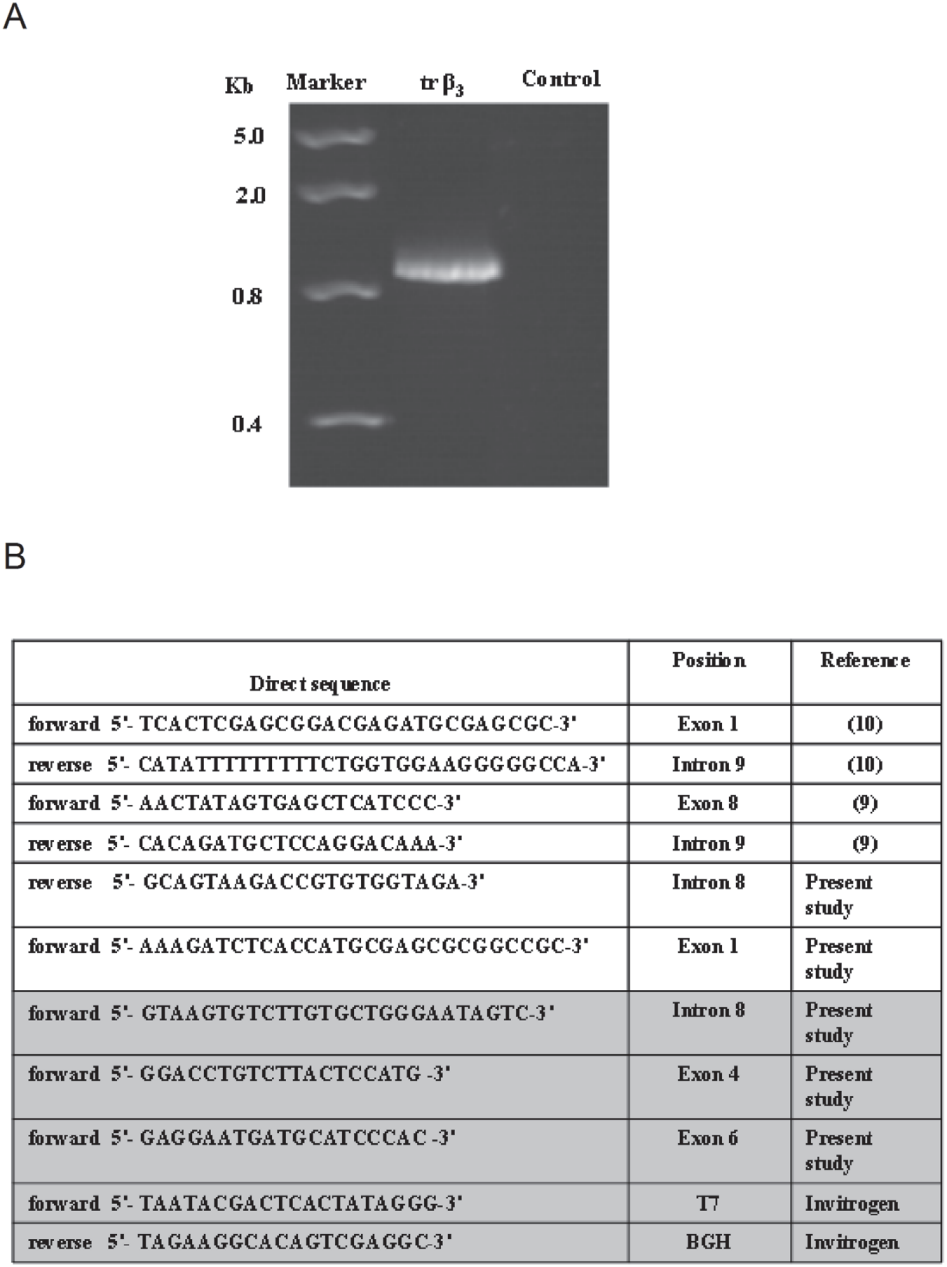
Characterization of sβ_3-_integrin detected in AML patient. (A) Total RNA was prepared from peripheral blood mononuclear cells (PBMCs) of leukemic patients and was reverse transcribed in cDNA. The sβ_3-_integrin sequence was amplified by PCR using specific primers. sβ_3-_integrin sequences (1201 bp) were detectable in AML patients. Negative controls were also used as described in Materials and Methods. (B) Primer sequences used in the amplification and sequencing of truncated β_3-_integrin. Primers used in the sequencing reactions are highlighted.

Previous studies showed that truncated alternative spliced β_3-_integrin can be secreted by tumour cells and deposited on the extracellular matrix [6]. To determine whether the sβ_3-_integrin detected in AML patient samples was generated by alternative splicing, trβ_3-_integrin coding sequence was amplified from cDNA patients’ samples using primers flanking the ATG initiation codon and the translation premature termination codon (intron 8) (Figure 2A–B). A sequence of 1201 bp was obtained. This novel trβ_3-_integrin sequence shows a unique intron 8 which differs from the wild type (wt) β_3-_integrin (Figure S1 A–C). This new intron 8 contains a premature stop codon which causes the lack of the transmembrane and cytoplasmic domain in the translated mature protein. These data indicate that the trβ_3-_integrin found in the plasmas of AML patients is produced by the alternative splicing and not a pseudogene or a degraded protein fragment.

### Soluble β_3-_integrin Increases NK Cell Proliferation

**Figure 3:**
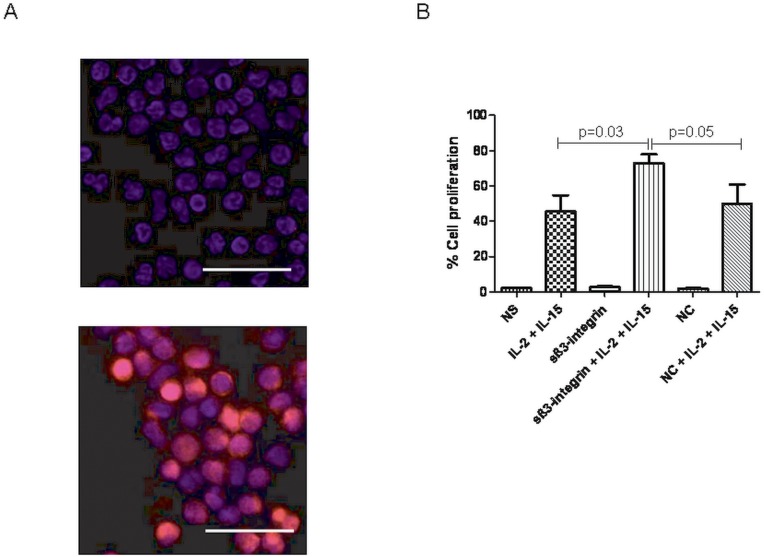
Soluble β_3_-integrin binds natural killer (NK) cells and induces proliferation. (A) NK cells were cultured without (upper picture) or with the His-tagged sβ_3_-integrin (lower picture). Protein binding was detected by fluorescence microscopy upon NK cell staining with a phycoerythrin-conjugated anti-His antibody. Diamidino phenylindol (DAPI) was used to stain the nucleus. Bar scale represents 25 µm. (B) Carboxyfluorescein succinmidyl ester (CFSE)-labelled NK cells were cultured in presence or absence of sβ_3_-integrin for 7 days. Figures represent the mean and standard deviation (mean±SD) of cell proliferation percentages of four independent experiments. Levels of significance were expressed as p-values (*p<0.05, **p<0.01, ***p<0.001).

NK cells are essential effector cells contributing for the reduction of the tumour burden [21], [22]. Therefore, it is important to evaluate the effect of proteins specifically detected in leukaemia patients in NK cell function. In this study, all functional assays were performed using recombinant sβ3_-_integrin which was produced as previously described [19]. Fluorescence microscopy analyses showed that sβ_3_-integrin is capable of binding NK cells (Figure 3A). We have investigated whether sβ_3_-integrin is capable of modulating NK cell proliferation. Interestingly, we have observed a significant increase in NK cell proliferation rates when these were cultured with IL-2, IL-15 and sβ_3_-integrin (73%±5%, p<0.05) in comparison to the cells cultured only with the cytokine cocktail (45.6%±9.2%). Also, sβ_3_-integrin-stimulated NK cell proliferation rates were superior than those observed after NK cell exposure to the cytokine cocktail and the control protein (49.8%±10.8%, ns). In the absence of cytokine cocktail, sβ_3_-integrin did not show an effect on the NK cell proliferation (1.9%±0.2%) compared to non-stimulated NK cells (2.2%±0.3%) (Figure 3B). These data suggest that sβ_3_-integrin may act synergistically with stimulating cytokines to enhance NK cell proliferation.

### Soluble β_3-_integrin Induces the Secretion of Pro-inflammatory Cytokines

Cytokines are crucial mediators and effector molecules during the immune response [23]. We have determined the effect of sβ_3-_integrin in the secretion of cytokines. Significant increases in the secretion levels of TNF-α (36.2±2.9 pg/mL, p<0.05) and IFN-γ (91.5±10.3 pg/mL, p<0.05) were detected upon NK cell culture in presence of sβ_3_-integrin in combination with IL-2 in comparison to the cytokine levels detected when the NK cells were only cultured in presence of IL-2 (TNF-α: 21.6±2.5; IFN-γ: 72.2±1.5 pg/mL). Stimulation of NK cells with sβ_3_-integrin in the absence of IL-2 did not affect the secretion levels of TNF-α or IFN- γ (Table 1). These data suggest that sβ_3_-integrin supports the secretion of pro-inflammatory cytokines which might have a direct cytotoxic effect or contribute to amplify the immune response.

**Table 1:** Natural killer)NK) cell cytokine secretion profile.

Analyte pg/ml	NC	IL-2	GPIIIa	GPIIIa + IL-2	KIR	KIR + IL-2
**TNF-α**	4.1±0.4	21.6±2.5	4.1±0.2	36.2±2.9 (*)	3.6±0.1	19.42±0.2
**IFN-γ**	3.2±0.0	72.2±1.5	3.2±0.0	91.5±10.3 (*)	3.2±0.0	61.1±9.4

Cytokine secretion was analysed in four independent experiments. Levels of significance were expressed as p-values (*p<0.05).

### Soluble β_3-_integrin Induces an Increase in Granzyme B Transcript Levels

**Figure 4:**
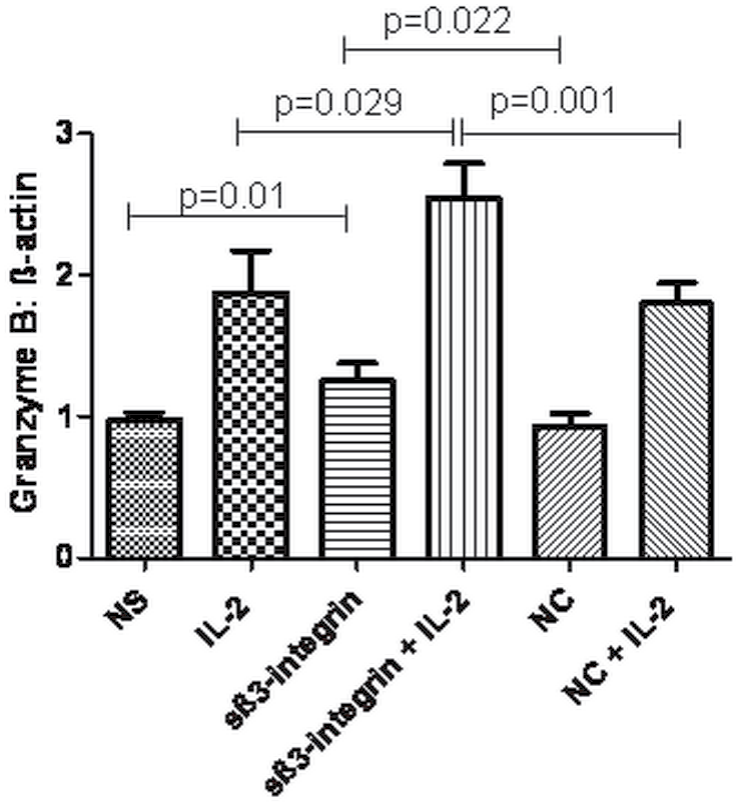
Soluble β_3_-integrin increases the cytotoxic potential of NK cells. Freshly isolated NK cells were cultured in presence or absence of 5µg/ml sβ_3_-integrin alone or in combination with IL-2 for 48 h. Non-stimulated NK cells or exposed to a control protein were used as controls. Granzyme B transcript levels were analysed by real time PCR. Housekeeping gene β-actin was used for normalization of cDNA levels. Figure depicts the mean and standard deviations (mean±SD) of values obtained in four separate experiments. Levels of significance were expressed as p-values (*p<0.05, **p<0.01, ***p<0.001).

Granzyme B is an important mechanism used by NK cells to induce the death of the target cells and it plays a crucial role in the control of tumor growth *in vivo* [24]. We have investigated whether sβ_3_-integrin may affect the cytotoxic potential of NK cells. Interestingly, significant higher Granzyme B transcripts levels were detected in sβ_3_-integrin-stimulated NK cells in presence of IL-2 (RQ: 2.6±0.2, p<0.05) in comparison to NK cells cultured with IL-2 alone (RQ: 1.9±0.3) or with the control protein in combination with IL-2 (RQ: 1.8±0.1). Also, NK cell stimulation with sβ_3_-integrin in the absence of IL-2 caused a significant increase of granzyme B transcript levels (RQ: 1.3±0.1) in comparison to non-stimulated NK cells (RQ: 1±0.1) or stimulated with the control protein (RQ: 1.8±0.1) (Figure 4). These results show that sβ_3_-integrin enhances the cytolytic potential of NK cells.

### Fas Ligand is Upregulated in NK Cells upon Soluble β_3-_integrin Stimulation

**Figure 5:**
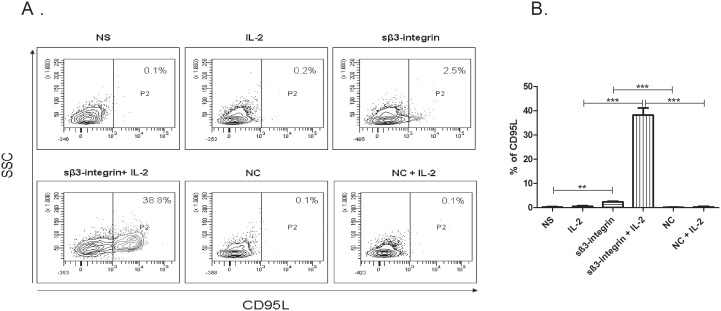
Expression of FasL is significantly upregulated upon sβ_3_-integrin exposure. Expression of FasL (CD95L) was detected on NK cells previously stimulated with 5 µg/ml sβ_3_-integrin alone or in combination with IL-2 for 48 h. Non-stimulated NK cells were also used as control. FasL expression levels were assessed by flow cytometric analyses. (A) Representative dotplots of FasL expression levels upon NK cell culture in the conditions described in Material and Methods. (B) The figure represents the mean and standard deviation (mean±SD) of FasL expression levels detected in four independent experiments. Levels of significance were expressed as p-values (*p<0.05, **p<0.01, ***p<0.001).

Fas ligand (FasL) causes apoptosis in Fas-expressing cells and serves as a major death-inducing factor in the immune response against tumor cells [25], [26]. In this study, we have observed a significant increase in FasL expression levels after exposure of NK cells to IL-2 in combination with sβ_3_-integrin (38.3%±2.9%, p<0.001) as compared to IL-2 alone (0.5.% %±0.3%) or in combination with a control protein (0.3%±0.2%) (Figure 5A). Similar to the effect on granzyme B, also NK cell stimulation with sβ_3_-integrin alone induced a significant upregulation of FasL expression (3.3%±0.3%, p<0.01) in comparison to non-stimulated NK cells (0.3%±0.2%) or stimulated with a control protein (0.2%±0.1%) (Figure 5B). Soluble β_3_-integrin showed to induce a strong upregulation of FasL on NK cells which may support their capacity to lyse leukemic cells.

### Soluble β_3-_integrin Supports NK Cell Cytotoxic Activity

**Figure 6:**
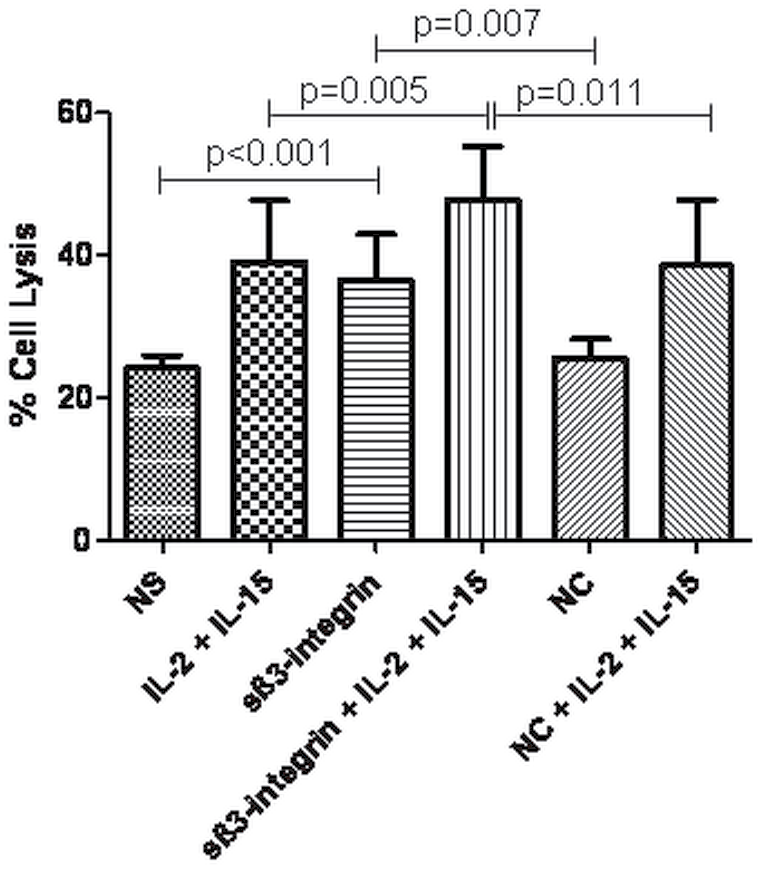
Soluble β_3_-integrin increases NK cell cytotoxic activity against K562 leukemia cells. Cytotoxic assays using NK cells previously cultured in the presence or absence of sβ_3_-integrin alone or in combination with IL-2 were exposed to primary K562 cells for 6 h at 5∶1 (effector:target) ratio. Target cell lysis was detected by flow cytometric analysis upon 7 aminoactinomycin (7-AAD) staining. (A) The figure shows the mean and standard deviation (mean±SD) of K562 cell lysis detected in four separated experiments. Levels of significance were expressed as p-values (*p<0.05, **p<0.01, ***p<0.001).

Above we have reported that sβ_3_-integrin promotes the secretion of cytotoxic cytokines and caused significant increases in granzyme B transcript levels as well as in FasL protein levels. To investigate the effect of sβ_3_-integrin in direct capacity of NK to lyse target cells we have performed cytotoxic assays using K562 cells as targets. Significantly higher target cell lysis rates were detected when NK cells exposed to a combination of IL-2 and sβ_3_-integrin (48.8%±7.5%, p<0.01) in comparison to IL-2 alone (39.1%±8.6%). Also, NK cell stimulation with sβ_3_-integrin alone resulted in a significant enhancement of target cell lysis (36.5%±6.5%, p<0.01) in comparison to non-stimulated NK cells (24.1%±1.8%) or stimulated with a control protein (25.6%±2.6%) (Figure 6). These data confirm that sβ_3_-integrin increases the capacity of NK cells to lyse their targets.

### Soluble β_3-_integrin Specifically Increases NK Cell Cytotoxicity against AML Blasts

**Figure 7:**
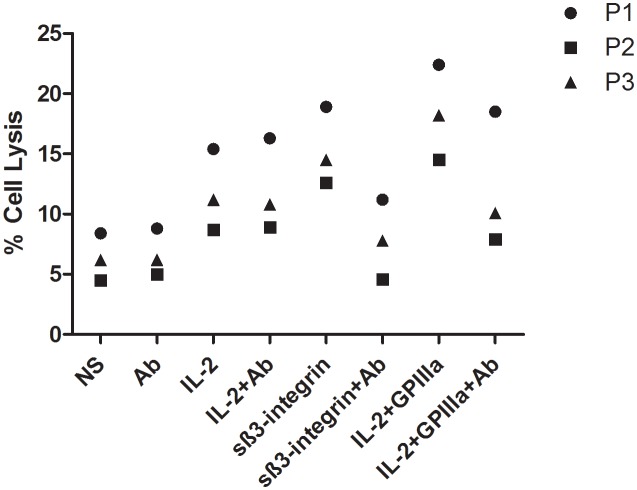
Soluble β_3_-integrin specifically increases NK cell cytotoxic activity against primary AML blasts. Cytotoxic assays using NK cells cultured in presence or absence of sβ_3_-integrin alone or in combination with IL-2 and a blocking antibody were exposed to primary AML blasts for 6 h at 5∶1 (effector:target) ratio. Target cell lysis was detected by flow cytometric analysis upon 7-AAD staining. The graph depicts NK cell lyse frequencies of AML blasts derived from three leukemic patients.

To confirm that the effect of sβ_3_-integrin also enhances NK cell cytotoxic activity against primary leukaemia cells, we have performed NK cell cytotoxic assays using primary AML blasts as targets. In addition, an antibody against sβ_3_-integrin was used to determine the specificity of the sβ_3_-integrin effect. NK cells stimulated with sβ_3_-integrin alone showed a significant increased in AML blast lysis rates (15.3%±2.6%, p<0.01) in comparison to non-stimulated NK cells (6.4%±1.6%) or when an antibody against sβ_3_-integrin was added to the culture (7.9%±2.7%). Furthermore, significant increases in AML blast cell lyses (18.4%±3.2%p<0.01) were observed when NK cells exposed to sβ_3_-integrin in combination with IL-2 in comparison to stimulation with IL-2 alone (11.8%±2.8%). Also this effect was abrogated by the addition of an anti- sβ_3_-integrin to the cultures (12.2%±4.6%, p<0.05) (Figure 7). These data demonstrate that sβ_3_-integrin-stimulated NK cells have a superior capacity to lyse primary AML blasts.

## Discussion

Usually β_3_-integrin is ectopically expressed as a membrane β_3_-unit associated with either α_v_ or α_IIb_ chains to form α_v_β_3_ or αIIbβ3 respectively. A number of alternatively spliced sequences for integrins subunits have been described [27]. Recently, studies have shown that these membrane-bound proteins play a crucial role in angiogenesis, platelet aggregation, and cancer metastasis [7]. In particular, sβ_3_-integrin has recently been identified in human prostate carcinomas, breast carcinomas, and melanoma cells [6]. In this study, a novel alternative sβ_3_-integrin transcript was detected in 35% of AML patients with an active form of the disease. Significantly higher sβ_3_-integrin levels were observed in AML patients prior chemotherapy compared with the sβ_3_-integrin levels observed after chemotherapy or in healthy individuals. The sβ_3_-integrin identified in this study resulted from the retention of 92 bp of intron 8, which is different from those previously described [6], [17]. Splicing of intron 8 did not cause a shift in the open reading frame, but it led to a premature TAG stop codon at 24 bp downstream from the end of exon 8. Unfortunately, we had no chance to check the molecular weight of the sβ_3_-integrin due to the limited plasma volume of the patients’ samples.

Unlike cytotoxic T lymphocytes (CTL), NK cells do not require antigen-specific recognition to lyse their targets [28]. Upon recognition of activating ligands on AML cells, NK cells contribute to lyse leukemic blasts through the secretion of proinflammatory cytokines such as IFN-γ and TNF-α, perforin or granzymes [16], [29], [30]. Granzyme B plays a major role in NK-mediated cytotoxicity by inducing the apoptosis of their targets [31]. In order to evade the immune response, AML cells secrete soluble factors such as TGF-β and IL-2R [32] that can inhibit both NK cell proliferation and cytotoxicity. Others immunosuppressive factors showed to impair the NK cell proliferation but not the cytolytic function [33]. Therefore, it is important to evaluate the effect of proteins specifically detected in leukaemia patients in NK cell function to determine their value as prognosis marker or therapeutic targets. Interestingly, we observed that sβ_3_-integrin is capable of binding NK cells. However, further studies are required to determine which receptors may be involved in the interaction of sβ_3_-integrin and NK cells. Nevertheless, our data demonstrated a synergistic effect between sβ_3_-integrin and IL-2 plus IL-15 in the induction of NK cell proliferation. Previous studies showed that the use of mature alloreactive NK cells has led to a shift in the paradigm of AML therapy [34]. Aiming at increasing the clinical advantage of allo-reactive NK cells, research is now focusing on the expansion of NK cells *in vivo* and *ex-vivo* [35]. Moreover, the feasibility of *ex-vivo* expanding of donor-derived NK cells populations after activation with soluble factors (e.g. IL-2) is currently being optimized and phase I clinical trials are ongoing [36], [37]. Therefore, it might be of clinical relevance to investigate the effect of sβ3-integrin in the expansion and functional properties of alloreactive NK cells. sβ3-integrin did not show the capacity to alter the natural cytotoxicity receptors (NCRs) or lectin-like receptor expression levels on NK cells (data not shown). Interestingly, sβ3-integrin alone or in combination with IL-2 showed to significantly induce the upregulation of granzyme B and FasL (CD95L) levels. These results indicated that sβ3-integrin may support the capacity of NK cells to induce apoptosis of their target cells. Granzyme B is critical in triggering apoptotic AML blasts released during granule exocytosis by NK cells [38]. FasL is intracellularly expressed in resting NK cells and it is translocated into the cell surface upon activation. FasL significantly contributes to the suppression of tumour growth *in vivo* [39].

NK cells may also induce the apoptosis of AML blasts and contribute to amplify the anti-tumour immune response through the release of proinflammatory cytokines such as IFN-γ and TNF-α [40], [41]. In cytokine secretion profiling analyses, sβ_3_-integrin promoted the secretion of IFN-γ and TNF-α by NK cells. To investigate the effect of sβ_3_-integrin on the capacity of NK to lyse leukemic cells, we performed cytotoxic assays using K562 and primary AML blasts cells as targets. When NK cells were exposed to sβ_3_-integrin in presence or absence of IL-2 for 48 hours, their cytotoxic activity against K562 cells and AML blasts was strongly increased. This finding suggests that sβ_3_-integrin supports NK cell cytotoxic activity against leukemic target cells. In antibody-mediated blocking experiments, we have shown that the effect of sβ_3_-integrin in NK cell cytotoxicity is specific. Antibody-mediated sβ_3_-integrin blocking showed to abrogate the enhanced capacity of NK cells to lyse primary AML blasts.

## Conclusions

Altogether, the results of this study suggest that sβ_3_-integrin is a strong activator of NK-cell cytotoxicity against tumour cells. To our knowledge, this is the first study that demonstrates the presence of a circulating alternative spliced sβ_3_-integrin at significantly increased levels in AML patients in the active state of the disease. Further studies will be required to evaluate the potential of using sβ_3_-integrin as a prognosis factor or biomarker for AML. Furthermore, sβ_3_-integrin may pave the way for the development of novel anti-leukemia therapies by supporting NK cells cytotoxicity against leukemic cells.

## Supporting Information

Figure S1 **Truncated alternative spliced β3-integrin sequence.** The sequence demonstrated that 1–1131 bp was identical to the wild type β3-integrin (GeneBank accession number: NM_000212) and that 1132–1234 bp was derived from intron 8 of β3-integrin gene (underlined). The termination codon is highlighted in bold characters. **B.** Predicted amino acid composition of the wild type (wt) and truncated (tr) β3 integrin protein. The protein sequence of the alternative spliced truncated β3-integrin was inferred from the cDNA sequence. The first 375 amino acid sequence of the tr β3-integrin is identical to the wt β3-integrin. The amino acids 376–382 of the tr β3-integrin were derived from intro 8 sequence (highlighted in bold letters). The transmembrane region of the wt β3-integrin is underlined. **C.** Electropherogram shows sequence of intron 8. The first 10 bp belong to exon 8 and then followed by 24 bp of intron 8 (underlined), which ends up with the premature stop codon (TAG).
(DOC)

Table S1 **Patient characteristics at diagnosis, among a total of 23 patients.**
(DOC)
