# Supplementary material for: Secreted β3-Integrin Enhances Natural Killer Cell Activity against Acute Myeloid Leukemia Cells
Source: PLoS One. 2014 Jun 11;9(6):e98936. doi: 10.1371/journal.pone.0098936 (PMC4053493; doi:10.1371/journal.pone.0098936)
Supplement: Figure S1 — Truncated alternative spliced β3-integrin sequence. The sequence demonstrated that 1–1131 bp was identical to the wild type β3-integrin (GeneBank accession number: NM_000212) and that 1132–1234 bp was derived from intron 8 of β3-integrin gene (underlined). The termination codon is highlighted in bold characters. B. Predicted amino acid composition of the wild type (wt) and truncated (tr) β3 integrin protein. The protein sequence of the alternative spliced truncated β3-integrin was inferred from the cDNA sequence. The first 375 amino acid sequence of the tr β3-integrin is identical to the wt β3-integrin. The amino acids 376–382 of the tr β3-integrin were derived from intro 8 sequence (highlighted in bold letters). The transmembrane region of the wt β3-integrin is underlined. C. Electropherogram shows sequence of intron 8. The first 10 bp belong to exon 8 and then followed by 24 bp of intron 8 (underlined), which ends up with the premature stop codon (TAG). (DOC) [file pone.0098936.s001.doc]

**Figure S1**

**A.**

| 0001 | ATGCGAGCGC | GGCCGCGGCC | CCGGCCGCTC | TGGGCGACTG | TGCTGGCGCT | GGGGGCGCTG |
| --- | --- | --- | --- | --- | --- | --- |
| 0061 | GCGGGCGTTG | GCGTAGGAGG | GCCCAACATC | TGTACCACGC | GAGGTGTGAG | CTCCTGCCAG |
| 0121 | CAGTGCCTGG | CTGTGAGCCC | CATGTGTGCC | TGGTGCTCTG | ATGAGGCCCT | GCCTCTGGGC |
| 0181 | TCACCTCGCT | GTGACCTGAA | GGAGAATCTG | CTGAAGGATA | ACTGTGCCCC | AGAATCCATC |
| 0241 | GAGTTCCCAG | TGAGTGAGGC | CCGAGTACTA | GAGGACAGGC | CCCTCAGCGA | CAAGGGCTCT |
| 0301 | GGAGACAGCT | CCCAGGTCAC | TCAAGTCAGT | CCCCAGAGGA | TTGCACTCCG | GCTCCGGCCA |
| 0361 | GATGATTCGA | AGAATTTCTC | CATCCAAGTG | CGGCAGGTGG | AGGATTACCC | TGTGGACATC |
| 0421 | TACTACTTGA | TGGACCTGTC | TTACTCCATG | AAGGATGATC | TGTGGAGCAT | CCAGAACCTG |
| 0481 | GGTACCAAGC | TGGCCACCCA | GATGCGAAAG | CTCACCAGTA | ACCTGCGGAT | TGGCTTCGGG |
| 0541 | GCATTTGTGG | ACAAGCCTGT | GTCACCATAC | ATGTATATCT | CCCCACCAGA | GGCCCTCGAA |
| 0601 | AACCCCTGCT | ATGATATGAA | GACCACCTGC | TTGCCCATGT | TTGGCTACAA | ACACGTGCTG |
| 0661 | ACGCTAACTG | ACCAGGTGAC | CCGCTTCAAT | GAGGAAGTGA | AGAAGCAGAG | TGTGTCACGG |
| 0721 | AACCGAGATG | CCCCAGAGGG | TGGCTTTGAT | GCCATCATGC | AGGCTACAGT | CTGTGATGAA |
| 0781 | AAGATTGGCT | GGAGGAATGA | TGCATCCCAC | TTGCTGGTGT | TTACCACTGA | TGCCAAGACT |
| 0841 | CATATAGCAT | TGGACGGAAG | GCTGGCAGGC | ATTGTCCAGC | CTAATGACGG | GCAGTGTCAT |
| 0901 | GTTGGTAGTG | ACAATCATTA | CTCTGCCTCC | ACTACCATGG | ATTATCCCTC | TTTGGGGCTG |
| 0961 | ATGACTGAGA | AGCTATCCCA | GAAAAACATC | AATTTGATCT | TTGCAGTGAC | TGAAAATGTA |
| 1021 | GTCAATCTCT | ATCAGAACTA | TAGTGAGCTC | ATCCCAGGGA | CCACAGTTGG | GGTTCTGTCC |
| 1081 | ATGGATTCCA | GCAATGTCCT | CCAGCTCATT | GTTGATGCTT | ATGGGGTAAG | TGTCTTGTGC |
| 1141 | TGGGAA**TAG**T | CCCGCGGAGA | GTCCACCTCA | TTTGGCTTAC | ACAGCAGGGC | TCAGATTTGT |
| 1201 | GAGTCCCAGT | TGCCAGTCTA | CCACACGGTC | TTA |  |  |

**B**

| wt- β_3_: 1  Tr **-** β_3_:1 | MRARPRPRPL  MRARPRPRPL | WATVLALGAL  WATVLALGAL | AGVGVGGPNI  AGVGVGGPNI | CTTRGVSSCQ  CTTRGVSSCQ | QCLAVSPMCA  QCLAVSPMCA | WCSDEALPLG  WCSDEALPLG |
| --- | --- | --- | --- | --- | --- | --- |
| wt- β_3_: 61  Tr **-** β_3_: 61 | SPRCDLKENL  SPRCDLKENL | LKDNCAPESI  LKDNCAPESI | EFPVSEARVL  EFPVSEARVL | EDRPLSDKGS  EDRPLSDKGS | GDSSQVTQVS  GDSSQVTQVS | PQRIALRLRP  PQRIALRLRP |
| wt- β_3_: 121  Tr **-** β_3_: 121 | DDSKNFSIQV  DDSKNFSIQV | RQVEDYPVDI  RQVEDYPVDI | YYLMDLSYSM  YYLMDLSYSM | KDDLWSIQNL  KDDLWSIQNL | GTKLATQMRK  GTKLATQMRK | LTSNLRIGFG  LTSNLRIGFG |
| wt- β_3_: 181  Tr **-** β_3_: 181 | AFVDKPVSPY  AFVDKPVSPY | MYISPPEALE  MYISPPEALE | NPCYDMKTTC  NPCYDMKTTC | LPMFGYKHVL  LPMFGYKHVL | TLTDQVTRFN  TLTDQVTRFN | EEVKKQSVSR  EEVKKQSVSR |
| wt- β_3_: 241  Tr **-** β_3_: 241 | NRDAPEGGFD  NRDAPEGGFD | AIMQATVCDE  AIMQATVCDE | KIGWRNDASH  KIGWRNDASH | LLVFTTDAKT  LLVFTTDAKT | HIALDGRLAG  HIALDGRLAG | IVQPNDGQCH  IVQPNDGQCH |
| wt- β_3_: 301  Tr **-** β_3_: 301 | VGSDNHYSAS  VGSDNHYSAS | TTMDYPSLGL  TTMDYPSLGL | MTEKLSQKNI  MTEKLSQKNI | NLIFAVTENV  NLIFAVTENV | VNLYQNYSEL  VNLYQNYSEL | IPGTTVGVLS  IPGTTVGVLS |
| wt- β_3_: 361  Tr **-** β_3_: 361 | MDSSNVLQLI  MDSSNVLQLI | VDAYGKIRSK  VDAYG**VSVLC** | VELEVRDLPE  **WE** 382 | ELSLSFNATC | LNNEVIPGLK | SCMGLKIGDT |
| wt- β_3_: 421  Tr **-** β_3_: 421 | VSFSIEAKVR | GCPQEKEKSF | TIKPVGFKDS | LIVQVTFDCD | CACQAQAEPN | SHRCNNGNGT |
| wt- β_3_: 481  Tr **-** β_3_: 481 | FECGVCRCGP | GWLGSQCECS | EEDYRPSQQD | ECSPREGQPV | CSQRGECLCG | QCVCHSSDFG |
| wt- β_3_: 541  Tr **-** β_3_: 541 | KITGKYCECD | DFSCVRYKGE | MCSGHGQCSC | GDCLCDSDWT | GYYCNCTTRT | DTCMSSNGLL |
| wt- β_3_: 601  Tr **-** β_3_: 601 | CSGRGKCECG | SCVCIQPGSY | GDTCEKCPTC | PDACTFKKEC | VECKKFDRGA | LHDENTCNRY |
| wt- β_3_: 661  Tr **-** β_3_: 661 | CRDEIESVKE | LKDTGKDAVN | CTYKNEDDCV | VRFQYYEDSS | GKSILYVVEE | PECPKGPDIL |
| wt- β_3_: 621  Tr **-** β_3_: 721 | VVLLSVMGAI | LLIGLAALLI | WKLLITIHDR | KEFAKFEEER | ARAKWDTANN | PLYKEATSTF |
| wt- β_3_: 781  Tr **-** β_3_: 781 | TNITYRGT |  |  |  |  |  |

**C.**

**
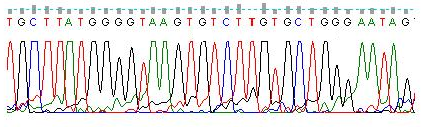
**
