# Supplementary material for: Secreted β3-Integrin Enhances Natural Killer Cell Activity against Acute Myeloid Leukemia Cells
Source: PLoS One. 2014 Jun 11;9(6):e98936. doi: 10.1371/journal.pone.0098936 (PMC4053493; doi:10.1371/journal.pone.0098936)
Supplement: Table S1 — Patient characteristics at diagnosis, among a total of 23 patients. (DOC) [file pone.0098936.s002.doc]

## Table S1. Patient characteristics at diagnosis, among a total of 23 patients

| **Characteristic** | **N (%)** |
| --- | --- |
| **Median age (range)** | 55 (25-72) years |
| **Sex, male/female** | 17/6 |
| **FAB Classification**  M0  M1  M2  M3  M4E_o_  M5  M6  M7  **Acute leukemia of ambiguous lineage** | 0 ( 0 )  4 ( 17.39 )  5 ( 21.74 )  0 ( 0 )  3 ( 13.04 )  1 ( 4.35 )  1 ( 4.35 )  0 ( 0 )  9 ( 39.13 ) |
| **WHO Classification**  **AML with multilineage dysplasia**  Following MDS  Without antecedent MDS (after CMML)  **AML not otherwise categorized**  AML, minimally differentiated  AML without maturation  AML with maturation  Acute myelomonocytic leukemia (AMMoL)  AMMoL with eosinophilia  Acute monoblastic leukemia  Acute erythroid leukemia  Acute megakaryoblastic leukemia  **Acute leukemia of ambiguous lineage** | 5 ( 21.74 )  1 ( 4.35 )  0 ( 0 )  4 ( 17.4 )  5 ( 21.74 )  0 ( 0 )  3 ( 13.04 )  1 ( 4.35 )  1 ( 4.35 )  0 ( 0 )  3 ( 13.03 ) |
